# Patient-prosthesis mismatch did not impact postoperative course and hemodynamics after biosthetic mitral valve replacement

**DOI:** 10.1186/1749-8090-8-S1-O277

**Published:** 2013-09-11

**Authors:** Hajime Ichimura, Tamaki Takano, Takamitsu Terasaki, Yuko Wada, Tatsuichiro Seto, Jun Amano

**Affiliations:** 1 Cardiovascular Surgery, Shinshu University, Matsumoto, Nagano, Japan

## Background

The clinical risk of patient-prosthesis mismatch (PPM) after mitral valve replacement (MVR) is still a matter of controversy. We investigated whether PPM in mitral bioprothesis affects the operative results or hemodynamic parameters.

## Methods

From 2004 to 2012, 34 patients underwent MVR with bioprosthesis in our institution. Patient’s age was 74±4 years old and male/female ratio was 23/11.

We implanted three valves; Epic Supra® (SJM, Minnesota) in 8 (25mm;5, 27mm;3), Mosaic® (Medtronic, Minneapolis) in 15 (25mm;5, 27mm;8, 29mm;2), Carpentier Edwards Perimount® (Edwards, Los Angeles) in 11 patients (25mm;1, 27mm;8, 29mm;2). Sixteen patients (group P) had small indexed effective orifice area (EOAI ≤1.2 cm2/m2), and the remaining 18 patients (group N) had acceptable EOAI. We compared operative results and hemodynamic parameters recorded by echocardiography between both groups. We also evaluated correlation between predicted EOAI and hemodynamic parameters after MVR.

## Results

Hospital mortality was 0% in both groups. Hospital stay was 26.5±15.6 days in group P and 34.2±20.7 in group N (p:NS). There was no difference between both groups in postoperative EF (p=0.92), max PG (p=0.38), mean PG (p=0.59), and measured EOAI from pressure half time (p=0.18). No correlation was found between predicted EOAI and measured EOAI (R2=0.0011), mean PG (R2=0.0000) and max PG (R2=0.0004).

## Conclusion

PPM did not affect operative results and postoperative hemodynamics in biopsoethetic MVR. Smaller valve might be alternative when large bioprosthesis is difficult to implant.

